# 9,10-Bis(iodo­ethyn­yl)anthracene

**DOI:** 10.1107/S2414314623005539

**Published:** 2023-07-04

**Authors:** Nehemiah Antoine, Marisa James, Keenan Dungey, Colin McMillen, Jared Pienkos

**Affiliations:** a Department of Chemistry and Physics, University of Tennessee at Chattanooga, Chattanooga, TN 37403, USA; b Department of Chemistry, Clemson University, Clemson, SC 29634, USA; University of Aberdeen, United Kingdom

**Keywords:** crystal structure, 9,10-bis­(iodo­ethyn­yl), anthracene, halogen bonding, π-stacking

## Abstract

The title compound, C_18_H_8_I_2_, is an ethynyl-substituted anthracene. The C—C—I bond angles deviate from 180°, being affected by inter­molecular I⋯π inter­actions. These inter­actions form a two-dimensional supra­molecular structure further supported by offset π–π stacking of neighboring anthracene moieties.

## Abstract 2

The title compound exhibits I⋯π halogen bonding and π-stacking in its extended structure.

## Graphical abstract

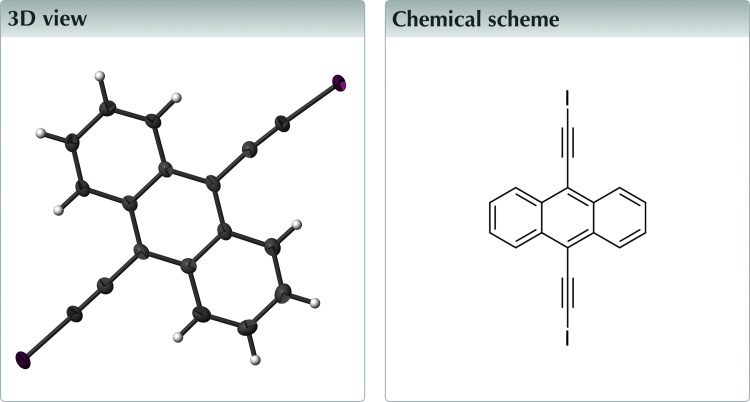

## Structure description

The flat, stable, and conjugated composition of anthracene derivatives makes them good candidates for two-dimensional mol­ecular crystals. Two-dimensional crystals can have unique properties with applications in electronics, biomedicine, and sensors (Yan *et al.*, 2023 ▸). The title compound is an iodoethynyl-substituted anthracene. The iodine functional groups provide the opportunity for halogen-bonding inter­actions. The synthesis and structure of the title compound are reported here.

**Figure 1:**
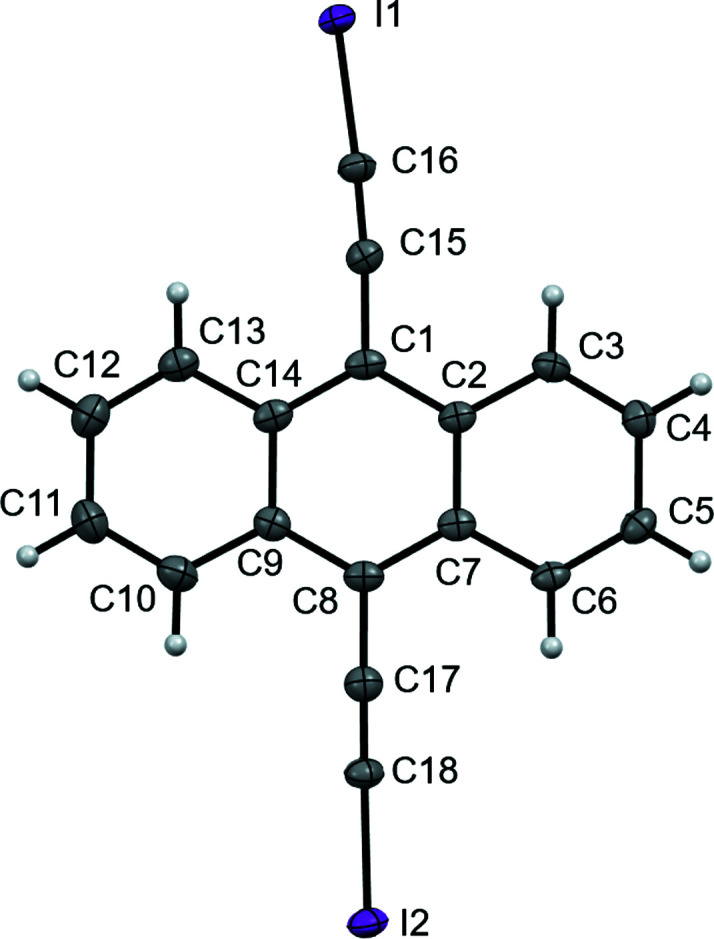
The title mol­ecule, showing the atom-labeling scheme and with displacement ellipsoids drawn at the 50% probability level.

**Figure 2:**
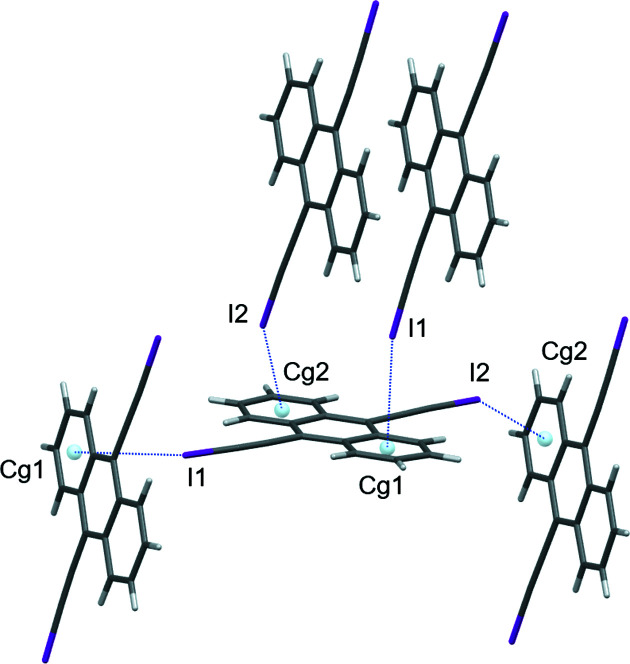
I⋯π inter­actions (blue dashed lines) occurring to and from a central mol­ecule of the title compound.

The crystal stucture represents the first example of an ethynyl–anthracene halogenated with iodine (Fig. 1 ▸). The C—I bonds have an average length of 1.996 (4) Å, similar to that found in 1,4-bis­(iodo­ethyn­yl)benzene [2.007 (7) Å; Barrès *et al.*, 2008 ▸], 4-iodo­ethnynylanisole [1.990 (3) Å; Dumele *et al.*, 2014 ▸], and other iodo­ethynyl derivatives (Lehnherr *et al.*, 2015 ▸). The 180° bond angle expected from the alkynyl C atoms and iodine, C15—C16—I1 and C17—C18—I2, are slightly bent to 177.4 (3) and 178.0 (3)°, respectively. This may be attributed to halogen bonding between C(*sp*)—I moieties and the π-electrons of the adjacent anthracene rings (Fig. 2 ▸), where I1 maintains its shortest I⋯centroid contact to the centroid of the C2–C7 ring (*Cg*1) [I1⋯*Cg*1 = 3.528 (4) Å and C16—I1⋯*Cg*1 = 151.2 (3)°] and I2 has a short contact to the centroid of the C9–C14 ring (*Cg*2) [I2⋯*Cg*2 = 3.767 (4) Å and C18—I2⋯*Cg*2 = 150.1 (3)°]. The bent nature of the C—I⋯centroid inter­actions leads to short I⋯C contacts ranging from 3.352 (4) to 3.655 (4) Å. The shorter contact between I1 and *Cg*1 appears to influence more significantly the bending of the entire alkynyl substituent [C1—C15—I1 = 173.8 (3)° *versus* C8—C17—I2 = 178.7 (3)°], notably pulling the I1 atom away from the central ring of the neighboring anthracene mol­ecule and toward its C2–C7 centroid.

**Figure 3:**
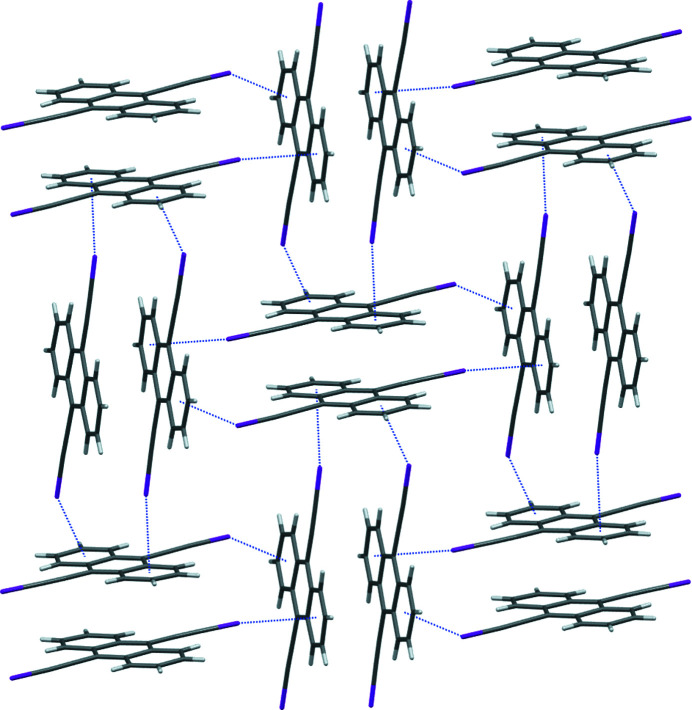
The two-dimensional supra­molecular motif formed *via* I⋯π inter­actions in the title compound.

**Figure 4:**
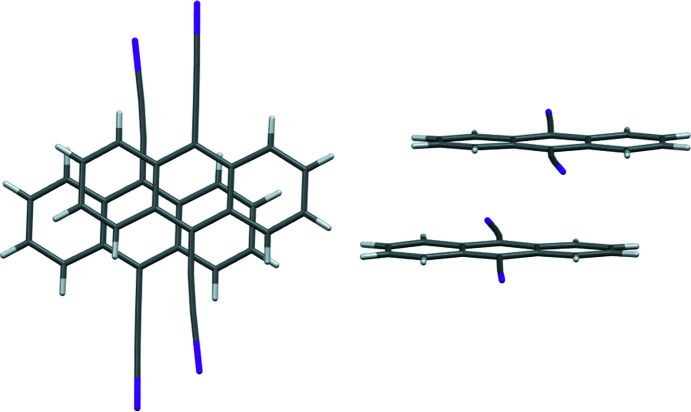
Top (left) and side (right) views of the offset stacking of neighboring mol­ecules in the title compound.

Propagation of the I⋯π inter­actions results in a two-dimensional supra­molecular structure in the (101) plane (Fig. 3 ▸). Inter­estingly, in 1,4-bis­(iodo­ethyn­yl)benzene, 4-iodo­ethnynylanisole, and 1-chloro-4-(iodo­ethyn­yl)benzene, the I⋯π inter­actions occur at a similar distance [for example, the shortest I⋯C contact is 3.427 (7) Å in 1,4-bis­(iodo­ethyn­yl)benzene, 3.392 (3) Å in 4-iodo­ethnynylanisole, and 3.417 (4) Å in 1-chloro-4-(iodo­ethyn­yl)benzene], but occur to the alkynyl C atoms rather than to the aromatic rings as in the title compound (Barrès *et al.*, 2008 ▸; Dumele *et al.*, 2014 ▸; Lehnherr *et al.*, 2015 ▸). The I atom in (*tert*-but­yl)[4-(iodo­ethyn­yl)phen­yl]carbamate (Kahlfuss *et al.*, 2016 ▸) does appear to inter­act with the aromatic system of a neighboring mol­ecule to form a one-dimensional I⋯π motif of similar C—I⋯centroid geometries to the title anthracene derivative. Anthracene portions of adjacent mol­ecules are arranged in an offset stacking arrangement (Fig. 4 ▸), with an inter­planar separation of 3.377 Å, a shortest C⋯C distance of 3.436 (5) Å, and a shortest centroid–centroid distance of 3.692 (5) Å. The inter­planar spacing of the anthracene scaffold in the title compound is shorter than in the offset stacking in 9,10-di­iodo­anthracene (3.602 Å; Peters *et al.*, 1996 ▸) and similar to that of the offset stacking in monoclinic 9,10-bis­(phenyl­ethyn­yl)anthracene (3.405 Å; Batsanov *et al.*, 2013 ▸).

## Synthesis and crystallization

The procedure was modeled after an analogous functionalization of an alkynylsilane (Tse *et al.*, 2021 ▸). 9,10-Bis(tri­methyl­silylethyn­yl)anthracene (0.0325 g, 0.0878 mmol), *N*-iodo­suc­cinimide (0.0531 g, 0.236 mmol) and AgNO_3_ (0.0022 g, 0.0129 mmol) were added to dry di­methyl­formamide (5 ml), and the resulting mixture was stirred under nitro­gen. After 5 h, the reaction mixture was diluted with EtOAc (30 ml) and washed with H_2_O (5 × 30 ml). The organic layer was dried *in vacuo*, resulting in an orange solid. The product was crystallized from the orange solid using vapor–vapor diffusion (CH_2_Cl_2_/hexa­nes).

^1^H NMR key spectroscopic features as determined from the crude product (400 MHz, chloro­form-*d*): δ 8.33 (*d*, *J* = 9.2 Hz, 4H), 7.82 (*m*, 4H).

## Refinement

Crystal data, data collection, and structural refinement details are summarized in Table 1 ▸.

## Supplementary Material

Crystal structure: contains datablock(s) I, global. DOI: 10.1107/S2414314623005539/hb4433sup1.cif

Structure factors: contains datablock(s) I. DOI: 10.1107/S2414314623005539/hb4433Isup2.hkl

Supporting information file. DOI: 10.1107/S2414314623005539/hb4433Isup3.cml

CCDC reference: 2191397

Additional supporting information:  crystallographic information; 3D view; checkCIF report

## Figures and Tables

**Table 1:** Experimental details

Crystal data	
Chemical formula	C_18_H_8_I_2_
*M*_r_	478.04
Crystal system, space group	Monoclinic, *P*2_1_/*n*
Temperature (K)	100
*a*, *b*, *c* (Å)	8.0022 (3), 15.0735 (7), 12.2506 (5)
β (°)	96.2749 (17)
*V* (Å^3^)	1468.83 (11)
*Z*	4
Radiation type	Mo *K*α
μ (mm^−1^)	4.27
Crystal size (mm)	0.17 × 0.15 × 0.13

Data collection	
Diffractometer	Bruker D8 Venture Photon 2
Absorption correction	Multi-scan (*SADABS*; Krause *et al.*, 2015 ▸)
*T*_min_, *T*_max_	0.774, 1.000
No. of measured, independent and observed [*I* > 2σ(*I*)] reflections	37187, 3373, 3084
*R*_int_	0.037
(sin θ/λ)_max_ (Å^−1^)	0.650

Refinement	
*R*[*F* ^2^ > 2σ(*F* ^2^)], *wR*(*F* ^2^), *S*	0.026, 0.063, 1.13
No. of reflections	3373
No. of parameters	181
H-atom treatment	H-atom parameters constrained
Δρ_max_, Δρ_min_ (e Å^−3^)	1.41, −0.90

Computer programs: *APEX3* (Bruker, 2017 ▸), *SAINT* (Bruker, 2016 ▸), *SHELXT2014* (Sheldrick, 2015*a* ▸), *SHELXL2016* (Sheldrick, 2015*b* ▸), *Mercury* (Macrae *et al.*, 2020 ▸) and *publCIF* (Westrip, 2010 ▸).

## full crystallographic data

### Crystal data

**Table:**

C_18_H_8_I_2_	*F*(000) = 888
*M**_r_* = 478.04	*D*_x_ = 2.162 Mg m^−^^3^
Monoclinic, *P*2_1_/*n*	Mo *K*α radiation, λ = 0.71073 Å
*a* = 8.0022 (3) Å	Cell parameters from 9890 reflections
*b* = 15.0735 (7) Å	θ = 2.7–30.6°
*c* = 12.2506 (5) Å	µ = 4.27 mm^−^^1^
β = 96.2749 (17)°	*T* = 100 K
*V* = 1468.83 (11) Å^3^	Column, red
*Z* = 4	0.17 × 0.14 × 0.13 mm

### Data collection

**Table:**

Bruker D8 Venture Photon 2 diffractometer	3084 reflections with *I* > 2σ(*I*)
Radiation source: Incoatec IµS	*R*_int_ = 0.037
φ and ω scans	θ_max_ = 27.5°, θ_min_ = 2.2°
Absorption correction: multi-scan (SADABS; Krause *et al.*, 2015)	*h* = −10→10
*T*_min_ = 0.774, *T*_max_ = 1.000	*k* = −19→19
37187 measured reflections	*l* = −15→15
3373 independent reflections	

### Refinement

**Table:**

Refinement on *F*^2^	Primary atom site location: dual
Least-squares matrix: full	Secondary atom site location: difference Fourier map
*R*[*F*^2^ > 2σ(*F*^2^)] = 0.026	Hydrogen site location: inferred from neighbouring sites
*wR*(*F*^2^) = 0.063	H-atom parameters constrained
*S* = 1.13	*w* = 1/[σ^2^(*F*_o_^2^) + (0.0142*P*)^2^ + 7.1388*P*] where *P* = (*F*_o_^2^ + 2*F*_c_^2^)/3
3373 reflections	(Δ/σ)_max_ = 0.001
181 parameters	Δρ_max_ = 1.41 e Å^−^^3^
0 restraints	Δρ_min_ = −0.90 e Å^−^^3^

### Special details

**Table:**

Geometry. All esds (except the esd in the dihedral angle between two l.s. planes) are estimated using the full covariance matrix. The cell esds are taken into account individually in the estimation of esds in distances, angles and torsion angles; correlations between esds in cell parameters are only used when they are defined by crystal symmetry. An approximate (isotropic) treatment of cell esds is used for estimating esds involving l.s. planes.

### Fractional atomic coordinates and isotropic or equivalent isotropic displacement parameters (Å^2^)

**Table:**

	*x*	*y*	*z*	*U*_iso_*/*U*_eq_	
I1	0.82384 (3)	0.70341 (2)	0.18275 (2)	0.02740 (8)	
I2	0.03465 (3)	0.12055 (2)	0.56404 (2)	0.02483 (8)	
C1	0.5433 (5)	0.4778 (2)	0.3413 (3)	0.0183 (7)	
C2	0.6141 (5)	0.4243 (2)	0.4292 (3)	0.0178 (7)	
C3	0.7841 (5)	0.4348 (3)	0.4760 (3)	0.0207 (7)	
H3	0.853257	0.477816	0.446422	0.025*	
C4	0.8486 (5)	0.3842 (3)	0.5624 (3)	0.0223 (8)	
H4	0.961959	0.392364	0.592776	0.027*	
C5	0.7470 (5)	0.3190 (3)	0.6078 (3)	0.0230 (8)	
H5	0.792490	0.284632	0.668972	0.028*	
C6	0.5867 (5)	0.3059 (2)	0.5643 (3)	0.0196 (7)	
H6	0.520936	0.261846	0.594840	0.024*	
C7	0.5139 (5)	0.3572 (2)	0.4729 (3)	0.0178 (7)	
C8	0.3466 (5)	0.3430 (2)	0.4245 (3)	0.0190 (7)	
C9	0.2770 (5)	0.3953 (2)	0.3353 (3)	0.0192 (7)	
C10	0.1094 (5)	0.3826 (3)	0.2846 (3)	0.0266 (8)	
H10	0.042007	0.337285	0.311230	0.032*	
C11	0.0440 (5)	0.4340 (3)	0.1990 (4)	0.0297 (9)	
H11	−0.067695	0.424074	0.166417	0.036*	
C12	0.1413 (6)	0.5018 (3)	0.1588 (3)	0.0282 (9)	
H12	0.094835	0.537029	0.098712	0.034*	
C13	0.3010 (5)	0.5178 (3)	0.2048 (3)	0.0234 (8)	
H13	0.363688	0.564785	0.177326	0.028*	
C14	0.3761 (5)	0.4645 (2)	0.2943 (3)	0.0190 (7)	
C15	0.6415 (5)	0.5464 (2)	0.2976 (3)	0.0201 (7)	
C16	0.7133 (5)	0.6047 (3)	0.2564 (3)	0.0226 (8)	
C17	0.2484 (5)	0.2737 (3)	0.4661 (3)	0.0224 (8)	
C18	0.1684 (5)	0.2157 (2)	0.5008 (3)	0.0227 (8)	

### Atomic displacement parameters (Å^2^)

**Table:**

	*U*^11^	*U*^22^	*U*^33^	*U*^12^	*U*^13^	*U*^23^
I1	0.02902 (14)	0.02681 (14)	0.02568 (14)	−0.00824 (10)	−0.00015 (10)	0.01045 (10)
I2	0.03161 (14)	0.01874 (12)	0.02455 (13)	−0.00641 (10)	0.00495 (10)	0.00300 (9)
C1	0.0253 (18)	0.0140 (16)	0.0175 (16)	−0.0007 (14)	0.0104 (14)	−0.0041 (13)
C2	0.0229 (18)	0.0134 (16)	0.0180 (16)	0.0009 (13)	0.0067 (14)	−0.0033 (13)
C3	0.0209 (18)	0.0204 (18)	0.0216 (18)	−0.0032 (14)	0.0061 (14)	−0.0020 (14)
C4	0.0230 (18)	0.0224 (19)	0.0211 (18)	0.0003 (15)	0.0007 (15)	−0.0044 (15)
C5	0.030 (2)	0.0184 (18)	0.0208 (18)	0.0028 (15)	0.0031 (15)	0.0004 (14)
C6	0.0267 (19)	0.0137 (16)	0.0193 (17)	−0.0010 (14)	0.0066 (14)	−0.0018 (13)
C7	0.0238 (18)	0.0136 (16)	0.0171 (16)	0.0001 (13)	0.0066 (14)	−0.0057 (13)
C8	0.0231 (18)	0.0151 (16)	0.0203 (17)	−0.0009 (14)	0.0094 (14)	−0.0038 (13)
C9	0.0209 (17)	0.0172 (17)	0.0200 (17)	0.0009 (14)	0.0052 (14)	−0.0049 (14)
C10	0.026 (2)	0.028 (2)	0.027 (2)	−0.0051 (16)	0.0061 (16)	−0.0027 (16)
C11	0.0226 (19)	0.034 (2)	0.032 (2)	0.0005 (17)	−0.0016 (16)	−0.0029 (18)
C12	0.033 (2)	0.028 (2)	0.0241 (19)	0.0056 (17)	0.0021 (16)	0.0009 (16)
C13	0.0274 (19)	0.0222 (19)	0.0216 (18)	0.0009 (15)	0.0078 (15)	−0.0011 (15)
C14	0.0268 (18)	0.0157 (16)	0.0158 (16)	0.0012 (14)	0.0086 (14)	−0.0034 (13)
C15	0.0234 (18)	0.0189 (18)	0.0188 (17)	0.0020 (14)	0.0055 (14)	−0.0018 (14)
C16	0.0266 (19)	0.0221 (18)	0.0197 (17)	−0.0043 (15)	0.0055 (15)	0.0016 (14)
C17	0.0251 (19)	0.0199 (18)	0.0225 (18)	0.0003 (15)	0.0046 (15)	−0.0029 (14)
C18	0.0274 (19)	0.0185 (18)	0.0228 (18)	−0.0048 (15)	0.0046 (15)	−0.0012 (14)

### Geometric parameters (Å, º)

**Table:**

I1—C16	1.996 (4)	C7—C8	1.420 (5)
I2—C18	1.996 (4)	C8—C9	1.412 (5)
C1—C14	1.413 (5)	C8—C17	1.433 (5)
C1—C2	1.413 (5)	C9—C10	1.428 (6)
C1—C15	1.437 (5)	C9—C14	1.434 (5)
C2—C3	1.426 (5)	C10—C11	1.362 (6)
C2—C7	1.431 (5)	C10—H10	0.9500
C3—C4	1.360 (5)	C11—C12	1.406 (6)
C3—H3	0.9500	C11—H11	0.9500
C4—C5	1.426 (5)	C12—C13	1.360 (6)
C4—H4	0.9500	C12—H12	0.9500
C5—C6	1.349 (6)	C13—C14	1.437 (5)
C5—H5	0.9500	C13—H13	0.9500
C6—C7	1.430 (5)	C15—C16	1.192 (5)
C6—H6	0.9500	C17—C18	1.190 (5)
			
C14—C1—C2	120.9 (3)	C7—C8—C17	119.3 (3)
C14—C1—C15	118.8 (3)	C8—C9—C10	122.3 (3)
C2—C1—C15	120.3 (3)	C8—C9—C14	119.4 (3)
C1—C2—C3	121.9 (3)	C10—C9—C14	118.3 (3)
C1—C2—C7	119.6 (3)	C11—C10—C9	121.5 (4)
C3—C2—C7	118.5 (3)	C11—C10—H10	119.3
C4—C3—C2	121.0 (3)	C9—C10—H10	119.3
C4—C3—H3	119.5	C10—C11—C12	120.1 (4)
C2—C3—H3	119.5	C10—C11—H11	119.9
C3—C4—C5	120.4 (4)	C12—C11—H11	119.9
C3—C4—H4	119.8	C13—C12—C11	121.0 (4)
C5—C4—H4	119.8	C13—C12—H12	119.5
C6—C5—C4	120.3 (4)	C11—C12—H12	119.5
C6—C5—H5	119.9	C12—C13—C14	120.8 (4)
C4—C5—H5	119.9	C12—C13—H13	119.6
C5—C6—C7	121.3 (3)	C14—C13—H13	119.6
C5—C6—H6	119.4	C1—C14—C9	119.7 (3)
C7—C6—H6	119.4	C1—C14—C13	122.0 (3)
C8—C7—C6	122.1 (3)	C9—C14—C13	118.2 (3)
C8—C7—C2	119.4 (3)	C16—C15—C1	175.4 (4)
C6—C7—C2	118.5 (3)	C15—C16—I1	177.4 (4)
C9—C8—C7	120.9 (3)	C18—C17—C8	179.3 (4)
C9—C8—C17	119.8 (3)	C17—C18—I2	178.0 (3)
			
C14—C1—C2—C3	178.2 (3)	C7—C8—C9—C10	179.9 (3)
C15—C1—C2—C3	−1.1 (5)	C17—C8—C9—C10	0.7 (5)
C14—C1—C2—C7	−1.6 (5)	C7—C8—C9—C14	−0.9 (5)
C15—C1—C2—C7	179.0 (3)	C17—C8—C9—C14	180.0 (3)
C1—C2—C3—C4	178.2 (3)	C8—C9—C10—C11	179.7 (4)
C7—C2—C3—C4	−1.9 (5)	C14—C9—C10—C11	0.4 (6)
C2—C3—C4—C5	0.3 (6)	C9—C10—C11—C12	−0.4 (6)
C3—C4—C5—C6	1.1 (6)	C10—C11—C12—C13	−0.5 (6)
C4—C5—C6—C7	−0.6 (5)	C11—C12—C13—C14	1.3 (6)
C5—C6—C7—C8	178.6 (3)	C2—C1—C14—C9	−0.5 (5)
C5—C6—C7—C2	−1.0 (5)	C15—C1—C14—C9	178.8 (3)
C1—C2—C7—C8	2.5 (5)	C2—C1—C14—C13	−179.9 (3)
C3—C2—C7—C8	−177.3 (3)	C15—C1—C14—C13	−0.5 (5)
C1—C2—C7—C6	−177.9 (3)	C8—C9—C14—C1	1.8 (5)
C3—C2—C7—C6	2.3 (5)	C10—C9—C14—C1	−178.9 (3)
C6—C7—C8—C9	179.1 (3)	C8—C9—C14—C13	−178.9 (3)
C2—C7—C8—C9	−1.3 (5)	C10—C9—C14—C13	0.4 (5)
C6—C7—C8—C17	−1.7 (5)	C12—C13—C14—C1	178.0 (4)
C2—C7—C8—C17	177.9 (3)	C12—C13—C14—C9	−1.3 (5)
